# Spinal Gout: A Rare but Serious Mimicker of Spinal Pathology—Report of Two Cases

**DOI:** 10.3390/reports8030135

**Published:** 2025-08-03

**Authors:** Muhammad Ishfaq, Rajeesh George, Rohan De Silva

**Affiliations:** Ng Teng Fong General Hospital, Singapore 609606, Singapore; devpura_acharige@nuhs.edu.sg

**Keywords:** gout, cord compression, facet joint cyst, anti-gout medications

## Abstract

In this report of two cases, we describe two patients with spinal involvement of gout. The first case involved a 67-year-old female who presented to the emergency department with a one-week history of weakness in both the upper and lower limbs, despite no prior history of gout. Cervical spine MRI revealed spinal cord compression at the C4 level from a posterior lesion. During surgery, chalky white deposits consistent with gouty tophi were observed in the ligamentum flavum within the epidural space at C4. These intraoperative findings correlated with elevated serum uric acid levels. The second case concerned a 68-year-old male who presented with a five-day history of right lower limb pain along with bilateral knee discomfort. Radiologic and laboratory evaluations revealed elevated inflammatory markers, negatively birefringent crystals in knee joint aspirate, spondylodiscitis at the L5-S1 level, and a right-sided synovial cyst at the T10–T11 level causing spinal cord compression. Following the initiation of anti-gout therapy, the patient experienced significant clinical improvement, normalization of inflammatory markers, and radiologic resolution of the thoracic synovial cyst.

## 1. Introduction

Gout is an inflammatory arthritis characterized by chronic hyperuricemia, leading to the deposition of monosodium urate (MSU) crystals in joints and connective tissues. It stands as the most prevalent crystal-induced arthropathy, with a global prevalence estimated at 0.08% [1]. While gout predominantly affects peripheral joints, involvement of the spine is considered rare [2]. Over the past two decades, the global incidence of gout has increased by approximately 63.44%, impacting both men and women. However, it remains more common in males, with a reported male-to-female ratio of 3:1, particularly in regions with a high sociodemographic index [3]. While genetic predisposition underlies much of gout susceptibility, the main drivers in recent years are poor diet, obesity, metabolic syndrome, increased alcohol intake, and aging populations. These risk factors have especially pushed gout prevalence upward in developed countries, and now in developing nations undergoing rapid lifestyle and dietary transitions [4]. Spinal gout often presents with symptoms such as back or neck pain, and elevated serum uric acid levels are frequently observed. The first documented case of spinal tophaceous gout was reported by Kersley et al. in 1950. Although uncommon, spinal gout can affect various components of the spinal anatomy, including the ligamentum flavum, epidural and intradural spaces, neural foramina, laminae, pedicles, and vertebral bodies. Such involvement can lead to a range of neurological manifestations, including radiculopathy, myelopathy, cauda equina syndrome, or neurogenic claudication, depending on the specific spinal segment affected.

Moreover, MSU crystal deposition in the facet joints may induce joint inflammation, cartilage degeneration, and soft tissue damage, potentially resulting in fluid-filled synovial cysts that exacerbate neurological symptoms. Unlike peripheral gout, which typically presents with recognizable clinical features, spinal gout often lacks classic signs, making diagnosis challenging and increasing the risk of misdiagnosis. This underscores the importance of heightened clinical suspicion, especially in atypical presentations [5,6].

## 2. Diagnostic Challenges

**Atypical presentation**: Spinal gout does not always manifest with the classical signs associated with peripheral gout, such as podagra. Its symptoms can mimic other conditions like spinal stenosis, disk herniation, or infections [7].

**Imaging limitations**: Conventional imaging modalities, including X-rays and MRI, may not effectively distinguish gouty tophi from other spinal pathologies. While CT scans can sometimes reveal calcified tophi, they are not definitive. Dual-energy computed tomography (DECT) has emerged as a valuable imaging tool in diagnosing and managing gout, particularly in identifying gouty tophi. DECT employs two different energy X-ray beams to differentiate materials based on their atomic composition, allowing for the specific identification of urate crystals [8]. Its sensitivity and specificity for gout are 90% and 83%, respectively, and it has been effective in diagnosing gout even in cases with negative synovial fluid analysis [9].

**Crystal studies**: The identification of monosodium urate crystals in synovial fluid or tophi is diagnostic of gout. However, these studies can sometimes yield negative results, possibly due to technical difficulties in obtaining samples from spinal locations or prior treatments altering the crystal composition [8].

**Biopsy and histopathology**: In many instances, a biopsy may be necessary to achieve a definitive diagnosis. Histopathological examination can reveal tophaceous deposits and chronic inflammatory changes consistent with gout. A high index of suspicion is required in suspected cases, and crystal study samples should be taken separately and not fixed in formalin solution and should be sent in normal saline [10].

Given the nonspecific symptoms and the potential for spinal gout to mimic other more common spinal conditions like disk herniation, spinal infections, and neoplastic lesions, maintaining a high index of suspicion is crucial [10,11,12]. This is particularly true for patients with a known history of gout or hyperuricemia, even if they do not present with typical peripheral joint involvement at the time of spinal symptoms. Additionally, while crystal studies can confirm the presence of monosodium urate crystals, these tests may sometimes yield negative results due to difficulties in obtaining spinal samples or previous treatments altering the crystal composition. In some cases, a biopsy may be necessary to achieve a definitive diagnosis, with histopathological examination revealing tophaceous deposits and chronic inflammatory changes consistent with gout [13,14].

## 3. Cases Presentation

### 3.1. Case 1

A 67-year-old woman with well-controlled diabetes mellitus, hypertension, hyperlipidemia, and peripheral vascular disease presented to the emergency department with a one-week history of worsening neck pain, along with numbness and weakness in both the upper and lower limbs. She was a non-smoker and non-alcoholic, and remained independent in her activities of daily living. She reported no urinary retention, bowel incontinence, headache, dizziness, fever, or history of gout.

On initial assessment, her vital signs were stable: blood pressure was 137/82 mmHg, body temperature was mildly elevated at 37.7 °C, and her Glasgow Coma Scale (GCS) score was 15. There was no spinal tenderness on examination.

Neurological assessment of the lower limbs showed bilateral weakness, more pronounced on the right side. Muscle power was graded 4/5 (Medical Research Council scale) in hip flexion, knee extension, and big toe dorsiflexion. Sensory examination was intact, and the digital rectal exam (DRE) was normal.

In the upper limbs, muscle power was reduced bilaterally to grade 3/5 in wrist dorsiflexion, shoulder abduction, and finger abduction. A positive Hoffmann sign and brisk reflexes in both the upper and lower limbs raised suspicion of cervical spinal cord compression.

Initial blood investigations revealed a white blood cell count of 15.13, an elevated erythrocyte sedimentation rate (ESR) of 85, and a C-reactive protein (CRP) level of 14.0, indicating inflammation. Bone metabolism tests were normal, but Vitamin D 25-Hydroxy levels were low.

Lumbar spine X-ray showed the loss of normal lumbar lordosis, grade 1 retrolisthesis of L3 over L4, moderate compression fractures at L4 and L5, and mild anterior wedging at L1 and L2. The orthopedic spine team assessed the patient and made a preliminary diagnosis of cervical myelopathy, recommending an MRI of the whole spine.

The following day, upper limb strength deteriorated further, prompting an urgent whole-spine MRI. Intravenous dexamethasone (8 mg stat, followed by 4 mg three times daily) was initiated, and the patient was kept nil per os (NPO) in preparation for potential emergency surgery. ASIA scoring was conducted every two hours, and a CT brain scan was requested to rule out a cerebrovascular event.

#### 3.1.1. Imaging Findings

CT Brain: Normal.

**Figure 1:**
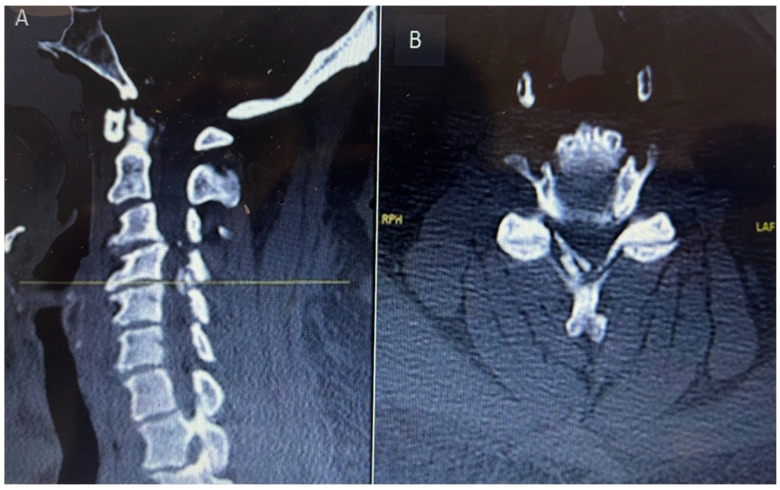
Preoperative sagittal (**A**) and axial (**B**) CT cervical spine showing calcific deposit at the cervical 4 level causing spinal stenosis.

CT C Spine: Ossification of the right-side ligamentum flavum noted at C4/C5 and C5/C6 levels, with associated spinal canal narrowing, more severe at C4/C5, as shown in Figure 1.

**Figure 2:**
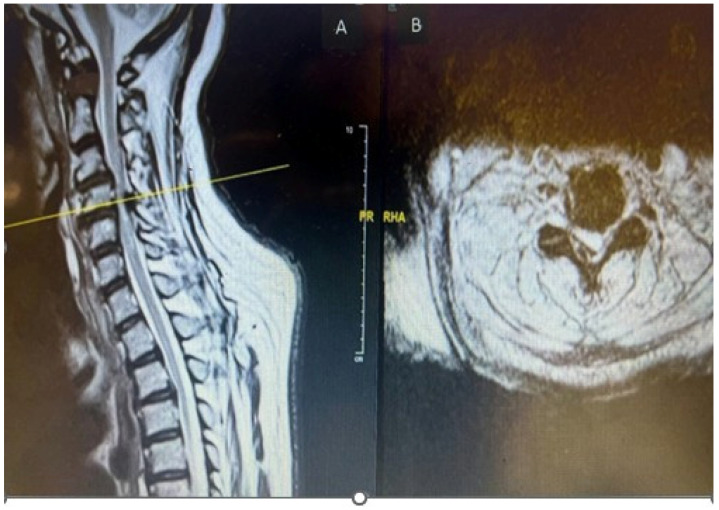
Preoperative MRI cervical spine T2-weighted sagittal (**A**) and axial (**B**) showing cord compression posteriorly with cord signal change at cervical 4 level.

MRI Whole Spine: Severe central canal narrowing causing compression of the spinal cord with myelomalacia at the C4/C5 level, with mild retrolisthesis at C4 over C5, thickening of the posterior longitudinal ligament, and ligamentum flavum hypertrophy. Various degrees of central canal narrowing and neural foraminal narrowing were noted at the C3/C4, C4/C5, C5/C6, and C6/C7 levels, with additional findings including a small synovial cyst at C6/C7, as shown in Figure 2A,B.

#### 3.1.2. Treatment and Management

The patient and her family were informed about her clinical condition and the MRI findings. Surgical management was planned, involving posterior cervical instrumentation from C3 to C5, along with decompressive laminectomy at the C4 and C5 levels. Preoperative intravenous antibiotics were administered. The posterior cervical spine was surgically exposed, and central decompression was achieved through complete laminectomy of C4 and C5. Lateral mass screw fixation was carried out from C3 to C5.

**Figure 3:**
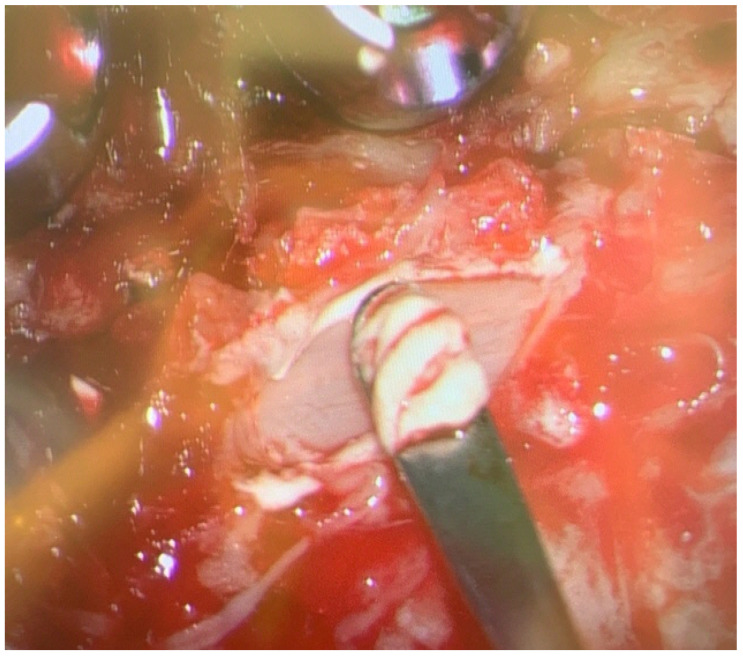
Intraoperative image showing chalky white deposit over the ligamentum flavum at cervical 4 level.

During the procedure, chalky white deposits were observed in the right-sided epidural space at the level of C4, overlying the ligamentum flavum, as shown in Figure 3. These deposits were removed in a piecemeal fashion, decompressing the spinal cord. Specimens were collected and sent for histopathological examination and culture. Baseline neuromonitoring showed weak somatosensory evoked potentials (SSEPs) and motor evoked potentials (MEPs) in both the upper and lower limbs, with no signs of nerve irritation detected on electromyography (EMG).

#### 3.1.3. Postoperative Findings

**Figure 4:**
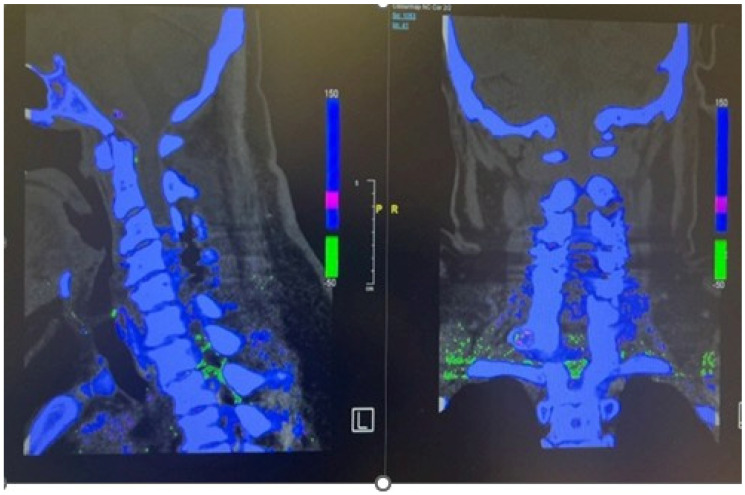
Postoperative dual-energy CT scan of cervical spine showing absence of green color urate crystals at the site of the surgery due to removal of gout tophi.

**Figure 5:**
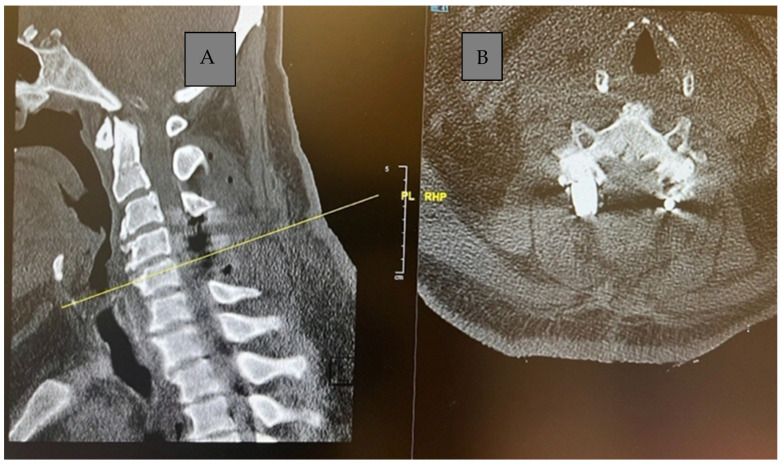
Postoperative CT scan of the cervical spine showing complete decompression at the cervical 4 and 5 levels. (**A**) Postop CT cervical spine sagittal view showing satisfactory decompression. (**B**) Postop CT cervical spine axial view showing posterior decompression and improvement in canal stenosis.

The chalky white deposits were sent for histopathological analysis; however, they were preserved in formalin, which precluded crystal analysis. Cultures taken from the epidural space showed no bacterial growth. Postoperatively, the patient’s serum uric acid level was elevated at 445 µmol/L (normal range: 150–370 µmol/L). She was referred to rheumatology for further management of hyperuricemia and commenced postoperative rehabilitation, which led to noticeable improvement in both upper and lower limb weakness. A dual-energy CT (DECT) scan of the cervical spine was performed to detect urate crystal deposition. However, the findings were inconclusive, likely due to the extensive removal of tophaceous material and thorough decompression during surgery as shown in Figure 4. Postoperatively, CT cervical spine was performed to check for sufficiency of boney decompression. Postop CT reported as satisfactory decompression, as shown in Figure 5A,B. Postop follow-up MRI cervical spine performed one and a half months later reported an improvement in spinal canal stenosis compared to the preop MRI.

### 3.2. Case 2

This 68-year-old male with a past medical history of obesity, diabetes mellitus, hypertension, chronic kidney disease 3b, knee osteoarthritis, psoriasis, ureteric calculus, and right-side meralgia paresthetica presented to the emergency department with the complaint of 3 days’ history of abdominal pain radiating towards the right lower back, which was worst on bending forward. There was associated foul-smelling urine without fever, frequency, dysuria, and hematuria. On examination, the patient was vitally stable, with generalized psoriatic rashes with negative bilateral renal punches and normal testicular examination. Laboratory tests showed a flat total white cell count with a neutrophil count of 7.10. A renal functions test was unremarkable. The urine dipstick result was suggestive of a UTI; therefore, the urine culture and sensitivity test were sent. X-ray KUB revealed a 0.5 cm radio density along the right hemi pelvis, likely a phlebolith as seen before on a prior CT with no calculus along the urinary tract. Degenerative changes were reported in the lumbar spine and left pelvis on X-ray of the lumbar spine, pelvis, and hip joints. The patient was discharged from the emergency department with orthopedics and spine follow-up.

**Figure 6:**
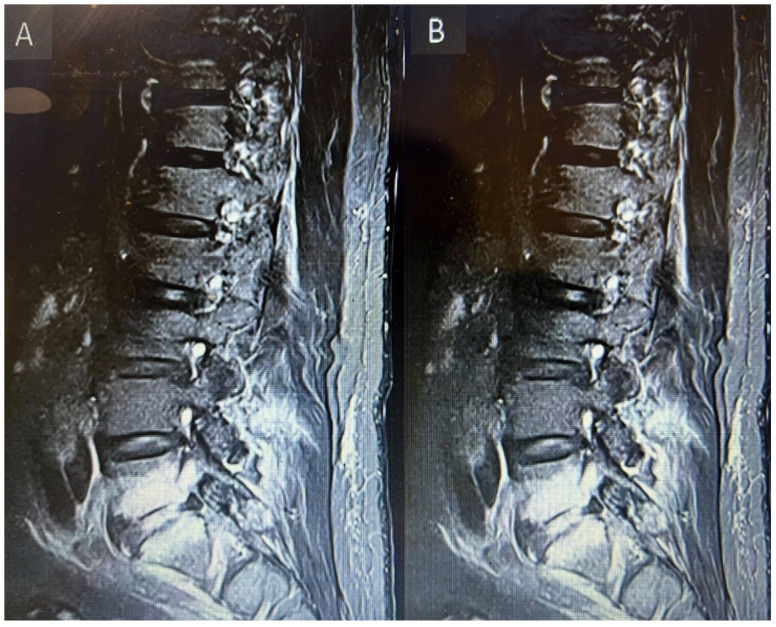
MRI lumbar spine short tau inversion recovery sequency sagittal views of first MRI before starting anti-gout medications (**A**) and follow-up MRI after starting anti-gout medications (**B**) showing spondylodiscitis at the level of lumbar 5, sacral 1 level.

**Figure 7:**
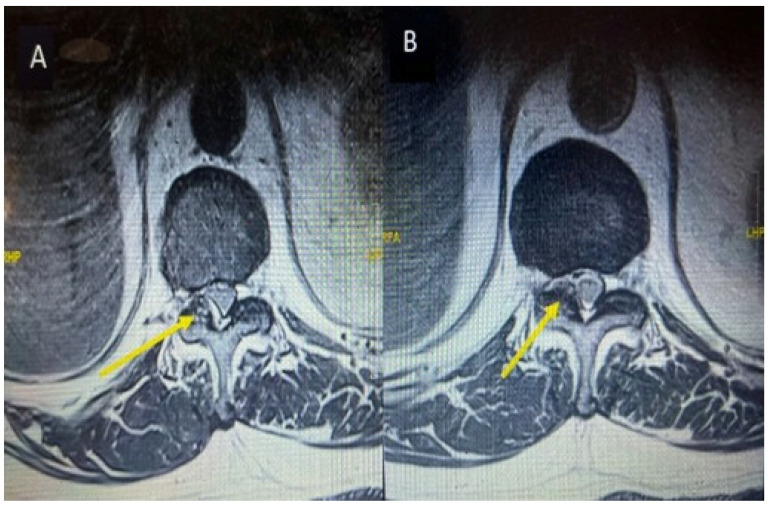
MRI thoracic spine T2-weighted at the level of T10/11 axial section before starting anti-gout medications (**A**) and axial section of follow-up MRI after starting anti-gout medications (**B**) showing a cyst on the right side as indicated by yellow arrow and total disappearance as shown by yellow arrow after starting anti-gout medications.

Two days after discharge from the emergency department, the patient again presented to the emergency department with the complaint of right iliac fossa pain for 1 day; therefore, after relieving pain, the patient was referred to general surgery. CT abdomen and pelvis, which was ordered by the general surgery team, reported a left stage horn calculus and left upper ureter inflammatory urethritis, with possible secondary minimal left pyelonephritis. He was referred to urology for further management. Subsequently, the patient developed right lower limb pain along the entire right thigh, posterior leg, and both surfaces of the right foot. There was also associated bilateral knee pain. There was 2/5 power in bilateral L2 and L3 myotomes limited by pain, with the rest of the neurological examinations unremarkable. The patient was referred to hip and knee and ortho spine. On the suggestion of the spine team, the primary team arranged an MRI lumbar spine with contrast and sent inflammatory markers. The MRI report revealed fluid within the right L5/S1 and edema in the adjacent L5/S1 vertebral bodies, suspicious for L5/S1 spondylodiscitis with osteomyelitis involving the right side more than the left. Other than this, at the same level L5/S1 posterior annular fissure and posterior disk protrusion causing moderate right exit foraminal narrowing abutting the right L5 exiting nerve root, as shown in Figure 6A. Additionally, a right-sided facet joint synovial cyst at T11/12 was noted with a size of 12 mm causing severe right-sided sub-articular stenosis compressing right T12 descending nerve root and moderate right exit foraminal narrowing abutting the right T11 exiting nerve root, as shown in Figure 7A.

**Figure 8:**
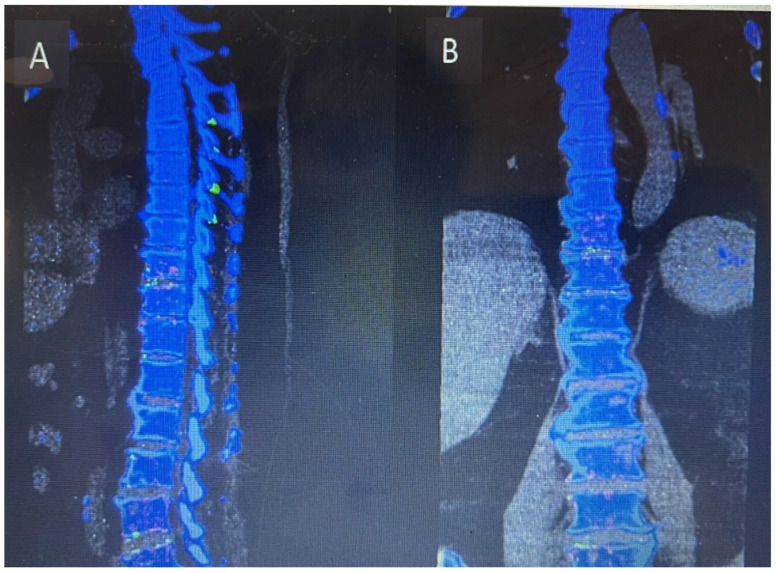
Dual-energy CT of whole-spine sagittal view (**A**) and coronal view (**B**) showing urate crystal deposits at the levels of T10/11 and L5/S1. The rest of the green pixelation is an artifact rather than urate crystals.

Inflammatory markers showed a raised CRP level of 292. Meanwhile, the hip and knee team advised X-rays of both knees reported as bilateral osteoarthritis with bilateral moderate to large suprapatellar effusion. Later on, they performed bilateral knee joints aspiration and sent the aspirate for laboratory evaluation, which showed raised nucleated cells (35,415), neutrophils (92%), and negatively birefringent crystals. Based on the knee joint aspiration result, the patient was referred to rheumatology for further assessment and serum uric acid was sent as per their suggestion, which showed a raised level of 532. The patient was counseled for image-guided biopsy of the reported levels on MRI spine in order to exclude infective spondylodiscitis and septic arthritis but due to patient reluctance, it was not performed. While keeping in mind the patient’s clinical, radiological, and lab test picture, a dual-energy CT whole-spine was performed, which reported urate deposits at the T10/11 and lower lumbar disks, especially at L5/S1. Green pixelation in the rest of the thoracolumbar spine appears to represent artifact rather than further urate deposits. In particular, there do not appear to be urate deposits at the region of the right T11/12 facet joint, as shown in Figure 8A,B. In order to start a definitive treatment, image-guided biopsy was discussed with the patient but not performed due to patient reluctance. Meanwhile, a multidisciplinary meeting was held between the infectious disease and rheumatology teams while keeping in mind the differential diagnosis of infective spondylodiscitis and facet cyst. The conclusion was made to treat the patient for gout based on his clinical features, laboratory tests, and radiological features. The rheumatology team started the patient on a 3 weeks’ tapering dose of prednisolone followed by 5 mg OD dose for a few months for gout prophylaxis. He was also started on allopurinol, an anti-gout medication, with a dose of 50 mg OD. Later on, the patient was transferred under the care of the rehabilitation team. The patient’s CRP and serum uric acid were trended over time and showed a progressive decline in their levels along with clinical improvement. Follow-up MRI lumbar and thoracic spine revealed improvement in the form of slight signal improvement at L5/S1, as shown in Figure 6B, and the disappearance of the right T10/11 facet cyst, as shown in Figure 8B. After completing rehabilitation, the patient was discharged to the home with the follow-up schedule with different concerned specialties.

## 4. Discussion

Gout is a metabolic disorder in which chronic hyperuricemia leads to monosodium urate crystal deposition occurring in joints. It either involves peripheral joints or the axial skeleton. Peripheral joint involvement including the first metatarsophalangeal joint, knee, ankle, and sometimes the first interphalangeal joint is more common than the axial skeleton [15,16]. Axial gouty arthropathy is less common but has more neurological consequences because of the involvement of the spine leading to axial spine pain, radiculopathy, myelopathy, cauda equina, and neurological deficit because of the compression of the nerve roots and spinal cord [16,17]. Elgafy H et al. in their systemic review documented that the lumbar spine is more commonly involved as compared to thoracic and cervical segments, but the most frequent symptoms are neck and back pain followed by symptoms of spinal cord compression, radiculopathy, fever, and atlantoaxial instability [18]. In our case series, there were only two cases, in which the first one had the involvement of the cervical spine with symptoms of neck pain and cervical myelopathy while the second one had the simultaneous involvement of lumbar and thoracic spinal segments with symptoms of low back pain and lumbar radiculopathy. Because of the rarity of gout involving the spine, especially the cervical spine, and the limited number of cases in our case series, it is difficult for us to calculate the frequency of the involvement of spinal segments and symptoms. Furthermore, statistical analysis is impossible with only two cases; therefore, studies with a larger number of cases are more helpful in this regard.

A literature review of spinal gout cases shows that the most common laboratory test finding is an increased uric acid level in the blood with a level of more than 7 mg/dl, which is the cut-off value for the definition of hyperuricemia. Most of the radiological findings on the plain X-rays and MRI spine are variable and nonspecific. MRI of tophaceous gout mostly shows low to intermediate intensity of T1-weighted sequence while T2-weighted images are highly variable, ranging from homogenous low to homogenous high intensity. MRI spine with contrast varies from homogenous to peripheral heterogenous enhancement. Because of the variable and nonspecific radiological picture of MRI, it is difficult to differentiate from the other mass-occupying lesions in the spinal cord. The most helpful radiological investigation is a dual-energy CT scan, which can differentiate it from other inflammatory mimicking conditions, especially rheumatoid arthritis, which is considered as a calcium phosphate deposition disease and the main reason is that it has two X-rays tubes with different peak kilovoltages (80 and 120 kVp), which can differentiate the urate crystals of gout from the calcium phosphate of rheumatoid arthritis. The sensitivity and specificity of dual-energy CT for monosodium urate crystals are 90% and 83%, respectively. Other than a dual-energy CT scan, cytological or histopathological studies can help to make a definitive diagnosis because of the high sensitivity and specificity for urate crystals [9,14,18]. In our case series, the level of blood uric acid was considerably high with a value of 445 mg/dL in the first case and 552 mg/dL in the second case, Consistent with the statement mentioned before. Similarly, we performed a dual-energy CT scan in both cases, which gave a more definitive clue of gout involvement of the targeted locations in the spine, which were detected by prior MRI images. The limitations in our study are that we were unable to perform the histological examination of the sample taken during the surgery because in the first case of the cervical spine, we took the sample for histological examination during the surgery but the sample was sent in formalin due to which it was not possible to perform a crystal examination while the second case was managed conservatively with the subsequent resolution of the thoracic spine facet joint cyst with anti-gout medications reported in the follow-up MRI spine. Based on these limitations, it is suggested that the tissue samples should be sent in normal saline in spite of fixing in formalin.

Spinal gout cases are treated either conservatively or surgically based on the presence or absence of neurological deficit. Pharmacological treatment is reserved for less severe cases without signs and symptoms of radiculopathy and myelopathy. Medications such as NSAIDS, colchicine, and corticosteroids are mainly used for symptoms relief in acute attacks without effecting the deposition of tophi in tissues and boney erosions. If the patient has a history of hypertension, heart disease, chronic kidney disease, gastric or duodenal ulcers, NSAIDS allergy, and receiving anticoagulant therapy then colchicine should be used in spite of NSAIDS. Anti-gout medications that are divided into first- and second-line medications are used to prevent further gout attacks with the goal of keeping the uric acid level less than 6 mg/dL. First-line anti-gout medications mainly reduce uric acid production, which include Xanthine oxidase inhibitors including allopurinol, oxypurinol, and febuxostat, and second-line medications are uricosuric agents that increase uric acid excretion, with sulfinpyrazone and probenecid on the list. Surgical treatment is indicated when there is spinal cord or nerve roots compression with neurological deficit. The main goal of the surgery is to decompress the spinal cord and nerves with the removal of tophi, which looks like a chalky white material [18]. We performed decompression of the cervical spine in our first case with the removal of chalky white material during the surgery because the radiological images were showing compression of the cervical spinal cord with progressive neurological deficit and the second case was managed conservatively because the patient gave a good response to anti-gout medications with an improvement in clinical symptoms, declining uric acid level, and complete resolution of thoracic spine facet cyst on follow-up MRI spine.

## 5. Conclusions

Spinal gout, though rare, is an important differential diagnosis in patients presenting with unexplained back or neck pain and neurological symptoms, especially among those with a history of gout or hyperuricemia. Due to its nonspecific presentation and diagnostic challenges—including the limitations of conventional imaging and crystal studies—maintaining a high index of suspicion is essential. Advanced imaging modalities like DECT and biopsy with histopathology can aid in accurate diagnosis and appropriate management; however, their limitations include cost, availability, and radiation exposure.

In non-surgical cases, to differentiate spinal gout from spondylodiscitis during image-guided biopsy, samples should be sent for crystal studies along with histopathology and culture with sensitivity testing. If the patient is unwilling to undergo image-guided biopsy, dual-energy CT (DECT) serves as a valuable non-invasive diagnostic tool with high sensitivity and acceptable specificity.

For patients presenting with neurological deficits who meet surgical criteria, tissue samples should be preserved in normal saline rather than formalin to maintain crystal structural integrity.

## Data Availability

Data results are stored in the electronic medical system of the hospital and may be requested from the corresponding author.

## Abbreviations

**GCS**	Glasgow Coma Scale
**DRE**	Direct rectal examination
**FBC**	Full blood count
**TW**	Total white cell count
**CRP**	C-reactive proteins
**ESR**	Erythrocyte sedimentation rate
**CT**	Computer tomography
**MRI**	Magnetic resonance imaging
**SSEP**	Somatosensory evoked potential
**EMG**	Electromyography
**MEP**	Magnetic motor evoked potential
**DECT**	Dual-energy computed tomography
**MRC scale**	Medical Research Council’s scale
